# Expression Levels of GHRH-Receptor, pAkt and Hsp90 Predict 10-Year Overall Survival in Patients with Locally Advanced Rectal Cancer

**DOI:** 10.3390/biomedicines11030719

**Published:** 2023-02-27

**Authors:** Dávid Fodor, Éva Pozsgai, Andrew V. Schally, Zoltán László, Éva Gömöri, Éva Szabó, László Rumi, Dorottya Lőcsei, Árpád Boronkai, Szabolcs Bellyei

**Affiliations:** 1 Department of Oncotherapy, Clinical Center, University of Pécs, Édesanyák Street 10, 7624 Pécs, Hungary; 2 Department of Public Health Medicine, Medical School, University of Pécs, Szigeti Street 12, 7624 Pécs, Hungary; 3 Department of Primary Health Care, Medical School, University of Pécs, Rákóczi Street 2, 7623 Pécs, Hungary; 4 Veterans Affairs Medical Center and South Florida Veterans Affairs Foundation for Research and Education, 201 NW 16th Street, Miami, FL 33125, USA; 5 Diagnostic, Radiation Oncology, Research and Teaching Center, Kaposi Somogy County Teaching Hospital Dr. József Baka, Guba Sándor Street 40, 7400 Kaposvár, Hungary; 6 Department of Pathology, Medical School, University of Pécs, Szigeti Street 12, 7624 Pécs, Hungary; 7 Department of Otorhinolaryngology, Clinical Center, University of Pécs, Munkácsy Mihaly Street 2, 7621 Pécs, Hungary; 8 Urology Clinic, Clinical Center, University of Pécs, Munkácsy Mihaly Street 2, 7621 Pécs, Hungary

**Keywords:** neoadjuvant radiochemotherapy, rectal cancer, Hsp90, GHRH-R, predictive markers, overall survival

## Abstract

Background: Rectal cancer constitutes nearly one-third of all colorectal cancer diagnoses, and certain clinical and molecular markers have been studied as potential prognosticators of patient survival. The main objective of our study was to investigate the relationship between the expression intensities of certain proteins, including growth-hormone-releasing hormone receptor (GHRH-R), Hsp90, Hsp16.2, p-Akt and SOUL, in specimens of locally advanced rectal cancer patients, as well as the time to metastasis and 10-year overall survival (OS) rates. We also investigated whether these outcome measures were associated with the presence of other clinical parameters. Methods: In total, 109 patients were investigated retrospectively. Samples of pretreatment tumors were stained for the proteins GHRH-R, Hsp90, Hsp16.2, p-Akt and SOUL using immunhistochemistry methods. Kaplan–Meier curves were used to show the relationships between the intensity of expression of biomarkers, clinical parameters, the time to metastasis and the 10-year OS rate. Results: High levels of p-Akt, GHRH-R and Hsp90 were associated with a significantly decreased 10-year OS rate (*p* = 0.001, *p* = 0.000, *p* = 0.004, respectively) and high expression levels of p-Akt and GHRH-R were correlated with a significantly shorter time to metastasis. Tumors localized in the lower third of the rectum were linked to both a significantly longer time to metastasis and an improved 10-year OS rate. Conclusions: Hsp 90, pAkt and GHRH-R as well as the lower-third localization of the tumor were predictive of the 10-year OS rate in locally advanced rectal cancer patients. The GHRH-R and Hsp90 expression levels were independent prognosticators of OS. Our results imply that GHRH-R could play a particularly important role both as a molecular biomarker and as a target for the anticancer treatment of advanced rectal cancer.

## 1. Introduction

Colorectal cancer is the third most common cancer in the world, and almost one-third of all of its diagnoses constitute rectal cancer [1]. The treatment protocol for rectal cancer depends primarily on the clinical stage of the cancer, with neoadjuvant radiochemotherapy (NRCT) followed by total mesorectal excision being the standard of care for patients with locally advanced rectal cancer [2]. NRCT includes the administration of 5-FU or capecitabine, which has been shown to downregulate the inhibitory immune checkpoint, cytotoxic T-lymphocyte protein 4 (CTLA-4), in colorectal cancer cells, thereby regulating the antitumoral immune response and possibly resulting in its therapeutic antitumor effect [3]. 

Patient survival and the 10-year cumulative incidence rate of distant metastasis have been shown to correlate with the response to NCRT [4], although this varies characteristically among individual patients. The histopathological tumor regression grade (TRG) has been reported to be an independent predictor of disease-free survival with which the pathological response to NRCT can be assessed [5,6,7]. 

In addition to the TRG, other clinical parameters such as poor condition at the time of operation and certain molecular markers, including the expression of matrilysin-2, have been shown to be predictive factors for overall survival (OS) [8,9]. For example, the lymphocyte-to-monocyte ratio (LMR) has been shown to correlate with survival in different cancer types, including colorectal and rectal cancers [10,11].

In an earlier study, we analyzed the expression levels of the growth-hormone-releasing hormone receptor (GHRH-R), heat shock protein (Hsp) 90, p-Akt, Hsp16.2 and heme-binding protein 2 (SOUL) in pretreatment rectal tumor samples, and found that GHRH-R and Hsp90 were independent predictive factors of the histopathological response to NRCT [12].

In addition to its neuroendocrine function, growth-hormone-releasing hormone (GHRH) acts as an autocrine/paracrine growth factor in various cancers, including gastrointestinal cancers [13,14]. The presence of GHRH-R and its splice variants—supporting the role of GHRH—has been demonstrated in different cancers, such as esophageal and colorectal neoplasms [15,16].

Heat shock proteins and their members with lower molecular weights, called small Hsp-s, are chaperone molecules that are expressed in response to different types of damage affecting the cells [17]. Hsp 90 is the most abundant Hsp, which chaperones 400 different client proteins [18]. Members of the Hsp90 family have a molecular weight range of 81–99 kDa, and are found in the cytosol, mitochondria, endoplasmic reticulum and nucleus of the cells and have multiple functions, including the autoregulation of the heat shock response, the folding of new proteins and influencing the cell cycle and cell proliferation [19]. The Hsp 90 family includes the constitutively expressed Hsp 90β and its stress-induced isoform, Hsp 90α, along with other members, TNF-receptor-associated protein1 (TRAP1) and glucose-regulated protein 94 (GRP94) [19]. Increasing evidence has demonstrated that Hsps, including small Hsp-s, play important roles in different types of tumors [20,21,22,23]. Hsp 90 has been shown to influence multiple signal pathways associated with cell cycle arrest and apoptosis, the regulation of invasion and metastasis and the sensitization of drug resistance [19,24,25,26]. Hsp 90 has been reported to be upregulated in colorectal cancer tissue compared to healthy colon tissues, which has led to investigations regarding the use of Hsp 90 inhibitors as potential therapeutic agents in colorectal cancer [19]. The novel Hsp90 inhibitor ganetespib, for example, has been found to inhibit the growth of colorectal cancer cell lines, to reduce colony formation and to inhibit the growth of tumors in vivo [27]. Furthermore, Hsp 90 inhibition has also shown promising results in a phase I trial of the single-agent ganetespib [19]. In neuroectodermal tumors, the level of the expression of a previously characterized small Hsp, Hsp 16.2, was found to correlate with the histological grades of different types of brain tumors [28,29,30].

Members of cytoprotective pathways and those involved in tumor necrosis have also been investigated as possible molecular targets in cancer research [31,32]. The PI3K/Akt pathway is a major antiapoptotic pathway and has been associated with the proliferation of malignant cells [33], while tumor necrosis has been shown to be an independent prognostic variable of cancer-specific survival [34]. A member of the BH3-domain-only protein family, heme-binding protein 2 (SOUL), was shown to facilitate necrotic cell death in oxidative stress through the permeabilization of the mitochondrial membranes [35,36].

In an earlier investigation, we analyzed the relationship between the expression of various proteins (Hsp 16.2, Hsp-90, GHRH-R and SOUL) and the response to NRCT, and found that GHRH-R and Hsp 90 were independent predictive factors of the histopathological response to NRCT [12]. The identification of proteins as potential biomarkers of outcomes is essential in order to individualize and increase the efficacy of treatment. Therefore, using our previous study as a basis, we recruited further patients to our study and followed up with them to investigate whether the mentioned proteins and additional clinical parameters could potentially be used as biomarkers for predicting patient outcomes.

In our present investigation, analyzing a larger patient sample than in our previous study [12], we aimed to investigate the relationship between the intensity levels of protein expression (GHRH-R, Hsp90, Hsp16.2, p-Akt and SOUL) in pretreatment tumor samples, as well as the 10-year overall survival (OS) and time to metastasis rates. It was also our objective to investigate whether the patients’ time to metastasis and 10-year overall survival (OS) rates were associated with certain clinical parameters (gender, time to surgery, tumor localization) of the patients.

## 2. Materials and Methods

### 2.1. Setting, Patients and Tumor Specimens

The study was carried out at a single site, at the University of Pécs Clinical Center, which has a dedicated cancer center (including Departments of Surgery and Oncotherapy) providing care for the South Transdanubia region of Hungary. Altogether, 114 patients with locally advanced (cT3/T4 or cN+ and cM0) rectal adenocarcinoma participated in our study (69 consecutive patients between January 2005 and December 2006 and 45 consecutive patients between January 2009 and March 2010). All patients received neoadjuvant radiochemotherapy (NRCT) followed by surgery. The pretreatment examinations included a digital rectal examination, sigmoidoscopy, biopsy, abdominal–pelvic CT, pelvic MRI, chest X-ray or CT. 

Here, 3D planned conformal radiotherapy was carried out in all cases with the belly board in the prone position, with 18 MV photons. The primary tumor as well as the lymph nodes at risk were covered with 3 irradiation fields and given 45 Gy-s in 25 fractions over a period of 5 weeks. As concomitant chemotherapy, 500 mg/m^2^ of 5-fluorouracil in a continuous infusion and a 30 mg/m^2^ folic acid bolus on days 1–5 of the 1st and the 5th weeks of radiotherapy were administered. Four weeks after the completion of NRCT, the patients were re-staged and a definitive surgical resection was performed 6–9 weeks after the neoadjuvant therapy in all 109 cases (5 patients were excluded from the study). A curative resection was performed in all cases. All patients gave informed consent, which was approved by the local ethics committee. The main clinical characteristics of the patients who underwent operations are given in Table 1.

### 2.2. Histopathological Evaluation

The rectal radiotherapy grading system adapted from the study by Mandard et al. [37] was used for the histological evaluation of the resected specimens to determine the pathological response to the neoadjuvant treatment. The five-point tumor regression grading (TRG) scale is based on the presence of residual tumor cells and the extent of fibrosis. The TRG includes the following: TRG1 (complete regression) is defined as the absence of residual tumor and fibrosis extending through the different layers of the rectal wall; TRG2 is characterized by the presence of rare residual tumor cells scattered throughout the fibrosis; TRG3 shows an increase in the number of residual tumor cells, but the fibrosis still predominates; TRG4 demonstrates residual tumor outgrowing the fibrosis; TRG5 is characterized by the absence of any tumor regression. In line with earlier studies to facilitate statistical analyses, the TRG sample was combined into two groups: good responders consisting of TRG1–2 and poor responders comprising TRG3–5 [37,38,39]. 

### 2.3. Preparation of Polyclonal Antibodies against Hsp16.2 and SOUL

Rabbits were immunized subcutaneously at multiple sites with 100 pg of recombinant Hsp16.2/GST and SOUL/GST fusion proteins, which were expressed as described previously [12,28,35] in Freund’s complete adjuvant. Four subsequent booster injections at 4-week intervals were given with 50 pg of protein in Freund’s incomplete adjuvant. Blood was collected 10 days after boosting, and the antiserums were stored at 20 °C. The IgGs were affinity-purified from sera by protein G-Sepharose chromatography (Sigma-Aldrich, St. Louis, MO, USA.), according to the manufacturer’s protocol. 

### 2.4. Immunohistochemistry

Sections of the pretreatment tumor tissue samples were fixed in formalin and embedded in paraffin. Subsequently, they were incubated with the following primary antibodies: GHRH-R primary antibody purchased from Abcam (Abcam Inc., Cambridge, MA, USA) (GHRH-R antibody detected the presence of both GHRH-R as well as the splice variants of the GHRH-R); p-AKT and total Hsp90 polyclonal rabbit primary antibodies purchased from Cell Signaling and Santa Cruz Biotechnology, Inc., Santa Cruz, CA, USA, respectively; and the self-developed anti-Hsp 16.2 and anti-SOUL polyclonal primary antibodies. The immunohistochemical staining was carried out according to the strepatavidin–biotin-peroxidase method with hydrogen peroxide/3-amino-9-ethylcarbazole development, using the Universal kit as previously described [40]. Only the secondary IgG was incubated with the control sections. The evaluation of the slides was performed with the help of an Olympus BX50 light microscope with an incorporated photography system (Olympus Optical Co., Hamburg, Germany). The staining intensity was recorded semiquantitatively as mild (+), moderate (++) or strong (+++), as described before [41]. For the internal positive control, the normal cellular and vascular structures of the samples were used. Positive areas around necrotic fields were excluded due to their probable stress-related upregulation. All slides were assessed by the same experienced pathologist blinded to the clinicopathological data.

### 2.5. Data Collection, Categorization and Outcome Measures

To increase the number of patients per group, the categories of the various variables were combined for these analyses: age over 60 years vs. 60 years or below; cT2 vs. cT3 vs. cT4; cN0 vs. cN1–2; distance from the anal verge of less than 5 cm versus between 5 and 10 cm versus more than 10 cm; time to surgery within 7 weeks versus over 7 weeks. For the statistical testing, the immunohistochemistry intensity values were dichotomized into low-intensity (0, +) and high intensity (++, +++) categories. The overall survival (OS) was defined as being from the date of diagnosis until the time of death from any cause. The time to metastasis was defined as being from the date of diagnosis until the documented first appearance of metastasis.

Data relating to patient parameters and survival were extracted from the clinic’s electronic medical database (eMedsol database) and the National E-health Infrastructure Database (EESZT). In cases where data regarding survival was not available from the above-mentioned databases, one researcher in the group contacted the patient or their family members for information. 

### 2.6. Statistical Analysis

The statistical analyses were performed with use of Statistical Package for the Social Sciences software 16.0 (SPSS, Chicago, IL, USA). A univariate Chi-square test was used to compare clinical parameters and biological markers for the tumor regression grade and clinical response. The relationship between the clinical and biological markers and the overall survival (OS) and time to metastasis rates were demonstrated using Kaplan–Meier curves, and the level of significance was determined using the log-rank test. Following the proportional hazard assumption testing of clinical and biological parameters found to significantly affect OS, a multivariate Cox regression analysis was performed (with a confidence interval of 95%) to test for the independent influence of potential prognostic factors on overall survival. In the course of the model estimation process, the “enter” method was used, meaning that all variables—excepting “tumor localization”—were used as possible covariants and included in the model, and their combined effect was analyzed. Probability (*p*) values < 0.05 were considered statistically significant, and the statistical tests were based on a two-sided significance level. 

## 3. Results

### 3.1. Protein Expression in Pretreatment Biopsy Specimens, Clinical Parameters and Histopathological Response to Therapy

The immunohistochemical evaluation of the pretreatment biopsy samples showed high-intensity staining (++, +++) for SOUL, Hsp 16.2, Hsp90, GHRH-R and for p-Akt in 60, 54, 67, 67% and 76% of the cases, respectively (Table 2).

A good pathological response was found in 52 and a poor response to NRCT was found in 57 cases out of the studied 109 specimens. 

High levels of GHRH-R expression in the pretreatment tumor specimens were significantly correlated with a poor histopathological response (*p* = 0.049), while no significant relationship could be detected between the expression of other proteins and the tumor regression grade (Table 3).

Patients whose time to surgery was more than 7 weeks had a significantly higher chance of having a good response to NRCT (*p* = 0.003) than those who underwent surgery within 7 weeks after NRCT. None of the other pretreatment clinical parameters was found to be significantly related statistically to the histopathological response (Table 4).

### 3.2. The Relationship between the Expression of Pretreatment Proteins and 10-Year Overall Survival (OS)

**Figure 1:**
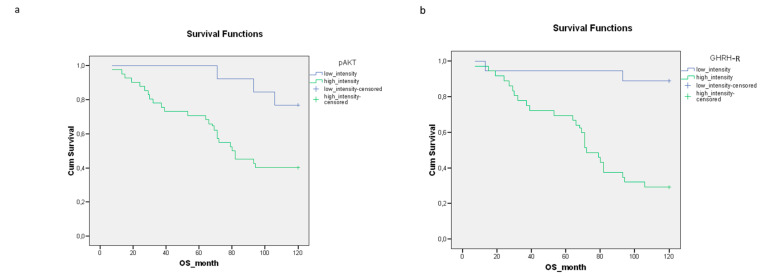

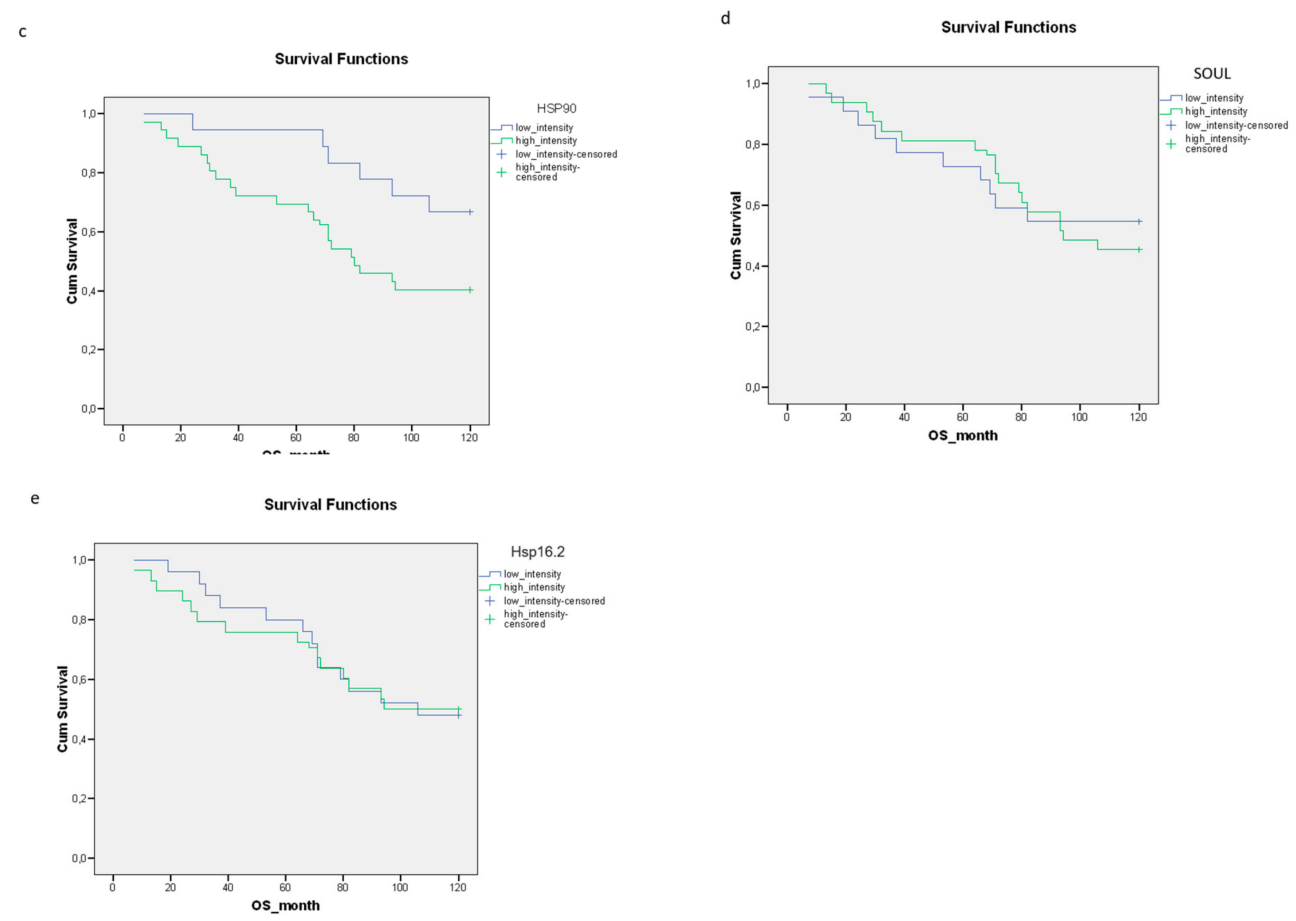
The relationship between the staining intensity of the pretreatment proteins (**a**) p-Akt (*p* = 0.001), (**b**) GHRH-R (*p* = 0.000), (**c**) Hsp90 (*p* = 0.004) (**d**) SOUL (*p* = 0.661) and (**e**) Hsp16.2 (*p* = 0.975) and the 10-year OS rate. The effects of biological markers on the overall survival were demonstrated using Kaplan–Meier curves and the level of significance was determined using the log-rank test. Probability (*p*) values < 0.05 were considered statistically significant.

We examined the relationship between pretreatment protein expression and 10-year OS. By 120 months, patients with tumors containing high levels of GHRH-R had a survival rate below 30%, compared to patients with cancers expressing low GHRH-R, where almost 90% of the patients were still alive. High levels of p-Akt, GHRH-R and Hsp90 were associated with significantly decreased 10-year OS rates (*p* = 0.001, *p* = 0.000, *p* = 0.004, respectively) (Figure 1a–c). The intensity of the SOUL and Hsp16.2 staining did not affect the 10-year OS rate significantly (Figure 1d,e).

### 3.3. The Relationship between Clinical Parameters (Histopathological Response, Tumor Localization, Gender) and 10-Year OS

**Figure 2:**
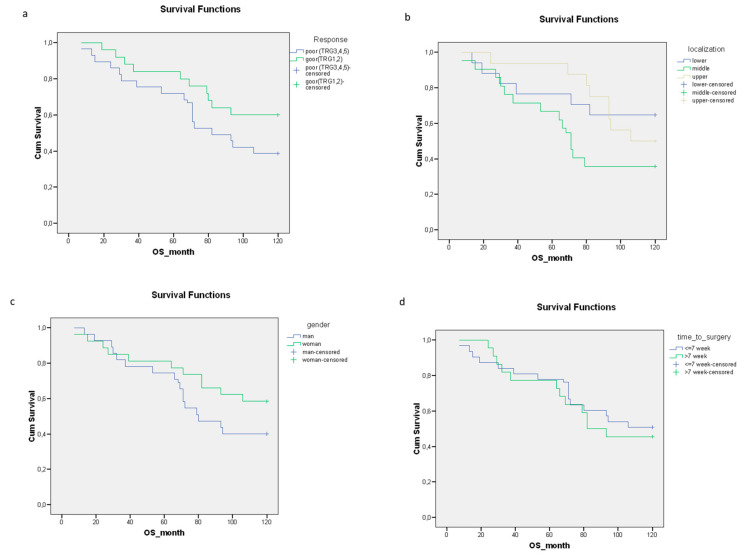
The relationships between clinical parameters including the (**a**) histopathological response (*p* = 0.029), (**b**) tumor localization (*p* = 0.015), (**c**) gender (*p* = 0.057) (**d**) time to surgery (*p* = 0.568) and 10-year OS rate. The effects of the clinical parameters on the overall survival were demonstrated using Kaplan–Meier curves and the level of significance was determined using the log-rank test. Probability (*p*) values < 0.05 were considered statistically significant.

We evaluated the effect of the individual clinical parameters on the 10-year OS rate. Good histopathological responses and lower-third tumor localization were associated with significantly improved 10-year OS rates, compared to patients with a poor histopathological response or upper- or middle-third tumors (*p* = 0.029; *p* = 0.015, respectively) (Figure 2a,b). Although the association was not significant, female gender was linked with an improved 10-year OS rate (Figure 2c). The time to surgery did not significantly affect the 10-year OS rate (Figure 2d).

The relationships between pretreatment proteins, clinical parameters and OS using the log-rank test are shown in Supplementary Table S1.

### 3.4. Analysis of the Expression of Pretreatment Proteins and Clinical Parameters as Potential Independent Prognostic Factors of Overall Survival

The biological and clinical markers found to have a significant association with 10-year OS were analyzed using a multivariate Cox regression analysis. Two biological markers, GHRH-R and Hsp90, were found to be significant independent prognostic factors of overall survival (GHRH-R: *p*: 0.000, EXP(B): −2.015, 95.0% CI: 0.044–0.401; Hsp90 *p*: 0.005, EXP(B): −0.928, 95.0% CI: 0.206–0.757) (Table 5).

A moderate correlation was found between p-Akt and GHRH-R, possibly explaining why p-Akt was not found to be a significant variable. However, when GHRH-R was excluded from the model, p-Akt remained significant (*p* = 0.000; Exp(B) = 0.204, 95.0% CI: 0.086–0.481), indicating that the prognostic role of p-Akt appears probable (data not shown). 

### 3.5. Relationship between the Expression of Pretreatment Proteins, Clinical Parameters and the Time to Metastasis

**Figure 3:**
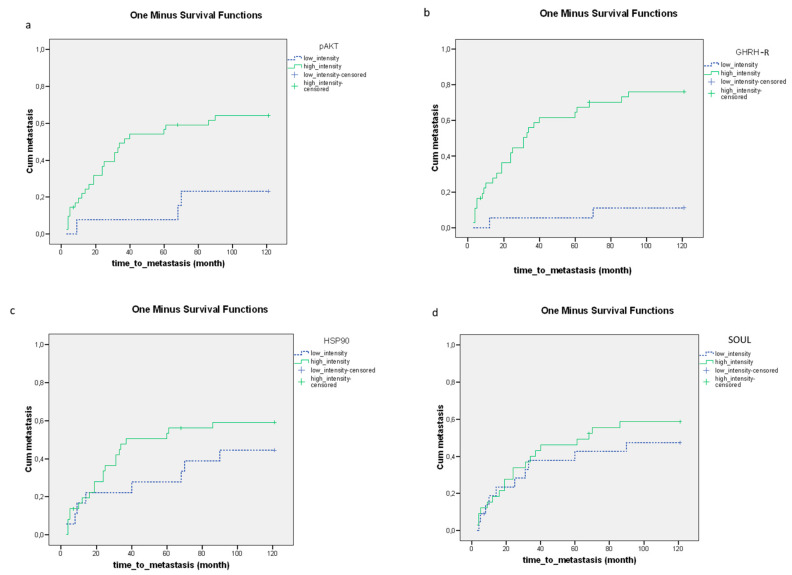

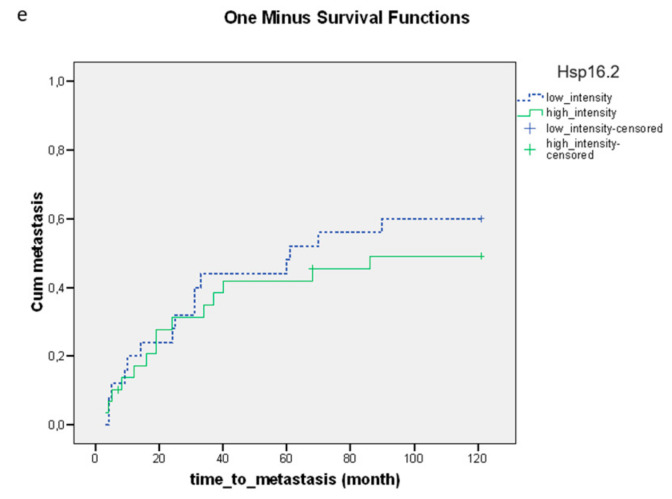
The relationship between the staining of the pretreatment proteins (**a**) p-Akt (*p* = 0.000), (**b**) GHRH-R (*p* = 0.000), (**c**) Hsp90 (*p* = 0.115), (**d**) SOUL (*p* = 0.310) and (**e**) Hsp16.2 (*p* = 0.328) and the time to metastasis. The effects of biological markers on the time to metastasis were demonstrated using Kaplan–Meier curves and the level of significance was determined using the log-rank test. Probability (*p*) values < 0.05 were considered statistically significant.

High expression levels of p-Akt and GHRH-R were associated with a significantly shorter time to metastasis (Figure 3a,b). No significant relationship could be detected between the expression levels of Hsp90, SOUL and Hsp16.2 and the time to metastasis (Figure 3c–e).

**Figure 4:**
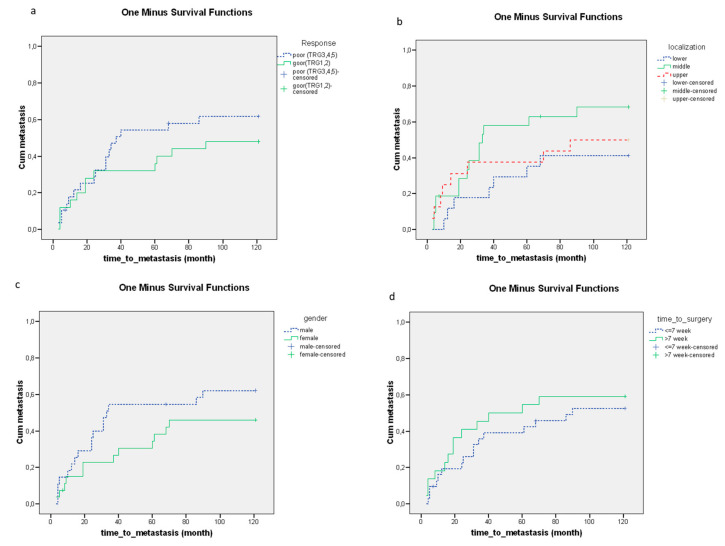
The relationships between clinical parameters including the (**a**) histopathological response (*p* = 0.170), (**b**) tumor localization (*p* = 0.048), (**c**) gender (*p* = 0.064), (**d**) time to surgery (*p* = 0.319) and time to metastasis. The effects of clinical parameters on the time to metastasis were demonstrated using Kaplan–Meier curves and the level of significance was determined using the log-rank test. Probability (*p*) values < 0.05 were considered statistically significant.

The tumors localized in the lower third of the rectum were associated with a significantly longer time to metastasis (Figure 4b). No significant relationship could be detected between the time to surgery, histopathological response, gender and time to metastasis (Figure 4a,c,d).

## 4. Discussion

In our study, we identified new molecular markers and analyzed previously investigated clinical markers as predictors of the time to metastasis and OS in patients with locally advanced rectal cancer.

Our previous study showed that the expression levels of GHRH-R and Hsp90 were independent predictive factors of histopathological response to NRCT [12]. In addition to the response to therapy, the appearance of the first metastasis and overall survival are both decisive outcomes for cancer patients. Supporting their possible role in locally advanced rectal cancer, we found that high levels of p-Akt, Hsp90 and GHRH-R were associated with significantly decreased 10-year OS, while increased staining intensities for p-Akt and GHRH-R were also linked to a significantly shorter time to metastasis. Malignant transformation and metastasis formation have been reported to be promoted by growth-hormone-stimulated insulin-like growth factor I (IGF-I) in different malignancies [42,43,44], and antagonists of GHRH have been shown to suppress the tumoral production of IGF-I and IGF-II by inhibiting the secretion of GH and blocking the binding of autocrine GHRH to receptors on cancer cells [45,46,47]. An earlier study reported that increased GHRH-R in tumor samples of gastric cancer correlated with poor overall survival and was an independent predictor of patient prognosis [48]. The overexpression of GHRH-R in colorectal malignancies has been detected previously [49]. Furthermore, the antagonization of GHRH has been shown to induce DNA damage in human colon cancer cells and subsequently to lead to p21-mediated cell cycle arrest and apoptosis [50]. Our findings indicate that GHRH-R, and consequently GHRH, may play an important part in both response to therapy and patient outcome measures. Although GHRH-antagonists have not yet been introduced into the clinical practice, the potential importance of GHRH-R both as a probable molecular target of therapy and—based on our present findings—as a predictor for survival in locally advanced rectal cancer is highly plausible.

Cell proliferation, migration and angiogenesis are processes that contribute to metastasis formation [49]. The levels of pAkt have been shown to be overexpressed in CRC compared to normal colorectal mucosa [51], while the angiogenesis of CRC was shown to be induced through the activation of the Akt/Erk signaling pathways [52]. Accordingly, in our tumor samples, intensive pAkt staining correlated with a shorter time to metastasis and decreased 10-year OS. The client proteins of Hsp 90 have also been known to be associated with the development and progression of cancer cells [53,54]. The four members of the Hsp90 family are encoded by genes that regulate tumor formation, adhesion, invasion, metastasis, angiogenesis and apoptosis [55]. Consequently, Hsp 90 may be overexpressed in tumors [56,57], as was shown in a number of reports [19], including a study that found a positive correlation between the overexpression of the mRNA for Hsp90 and metastasis and poor prognosis in CRC [58]. Hsp90 has been found to facilitate the progression of cancer cells by increasing the activity of oncogenic protein kinases, including MAPK, JAK2/STAT3 pathways and the previously mentioned PI3K/Akt signaling pathway, which is responsible for promoting cell proliferation and survival and is often overactivated in cancer cells [25]. In line with these reports, we found high levels of Hsp 90 in our rectal cancer specimens, and our results indicated that high levels of Hsp 90 levels in the pretreatment rectal cancer specimens were associated with a worse prognosis and decreased 10-year OS. Along with GHRH-R, Hsp90 was found to be an independent prognosticator of OS in our study. Based on our findings and previous evidence, it appears plausible that the elevated levels of Hsp90 in the cells of our rectal tumor samples may have promoted the PI3K/Akt signaling pathway and thereby increased the pAkt levels in the tumor tissue samples, which ultimately resulted in our finding that increased levels of both pAkt and Hsp90 were associated with tumor progression and subsequently significantly poorer 10-year survival. 

Since Hsp-s have been detected in extracellular vesicles in various biological fluids of cancer patients, the recent research has turned to investigating them as potential sources of biomarkers [59]. The increase in Hsp70 in the serum, for example, has been reported to be associated with the stage of the colorectal cancer, while high levels of soluble Hsp70 were reported to be associated with poor survival [60,61]. Hsp 90AA1/HSP90alpha expression was also shown to be significantly higher in the serum of CRC patients compared to healthy individuals [56]. On one hand, these results support our findings of the prognostic relevance of Hsp 90, while on the other hand, to our knowledge, our study is the first to report the significant association between the intracellular Hsp90 expression of rectal cancer samples and a poor 10-year prognosis. Since immunohistochemical testing remains a comparatively easy and cheap method and histopathological testing remains a standard part of tumor diagnostics, our study may have relevance from a practical point of view, whereby the intracellular testing of tumor tissues for Hsp90 and GHRH-R could potentially be used routinely and easily in clinical practice, if further validating research is conducted.

Although Hsp16.2 has been shown to inhibit cell death by binding to Hsp90, and through the activation of the PI-3kinase/Akt pathway [30] and was also been found to be overexpressed in esophageal cancer [62], the intensity of the Hsp 16.2 staining showed no significant relationship with either the time to metastasis or 10-year OS in our present study. Furthermore, both Hsp16.2 and SOUL were predictors of a negative response to NRCT in esophageal cancer according to a previous report [63], yet neither protein correlated with the survival rate in our patients. Thus, it is plausible that in contrast to esophageal cancer cells, the main manner of tumor development in rectal cancer cells does not occur through the Hsp16.2-mediated antiapoptotic or SOUL-mediated necrotic pathways; however, the exact molecular mechanisms require further investigation.

Among the studied clinical parameters, the time to surgery was significantly associated with the response to therapy but did not affect the time to metastasis or 10-year OS in our study. Our findings are supported by other recent studies on rectal cancer patients in which no significant differences in disease-free or overall survival were found between patients with different time intervals to surgery [64,65,66]. Furthermore, according to earlier reports, a longer than 6–8-week period between the completion of NRCT and surgery was associated with a significantly higher rate of response [67,68]. This may be explained by the phenomenon where biological changes induced by radiation need time to develop. 

In recent years, several investigations have supported the prognostic role of TRG in locally advanced rectal cancer, where the degree of tumor regression following NRCT, indicating the histopathological response to NRCT, was found to correlate with the cumulative incidence rate of distant metastasis, disease-free survival and overall survival [4,7]. Corresponding to these reports, we also observed that a good histopathological response leads to a significantly improved 10-year OS. 

There have been conflicting reports regarding the role of tumor localization in locally advanced rectal cancer patients. While an earlier study reported that a distance from the anal verge of more than 5 cm was associated with significantly lower downstaging rates [69], a more recent but smaller study discovered that a distance of more than 5 cm was related to a significantly higher complete pathological response rate and improved 5-year survival [70]. However, a very recent meta-analysis showed that cancers closer to the anal verge were significantly more likely to achieve a complete pathological response [71]. In accordance with this large analysis, we found that a distance of less than 5 cm from the anal verge, i.e., lower-third localization, was related to a longer time to metastasis and increased 10-year OS. These findings may be explained by the clinical behavior of lower rectal cancers. The tumors in the lower third of the rectum can be reached more easily by physical examination and signs of bleeding may also be apparent sooner to the patient than tumors in the middle or upper third of the rectum, thereby possibly leading to earlier detection of the tumor. 

Another clinical parameter that has been investigated as a potential prognostic factor is the gender of the patient. Females have been found to have significantly better OS in CRC than males [71], a tendency that we also observed among our patients, although the advantage was not found to be significant among our patients. 

Although the parameters investigated as potential clinical prognosticators mostly supported the results from previous studies, our results highlight the possible importance of utilizing relatively easily accessible information, such as the localization of the tumor in everyday practice.

Our study had certain limitations. This was a comparatively small study, and further potentially relevant clinical information (e.g., BMI, smoking status), possibly influencing our results, was not collected about the patients. In addition, since this was a preliminary study, it lacked precision in and an individualized approach to patient enrollment, which are prerequisites of a clinical trial. Thus, further research with larger patient samples and more accurately designed and conducted clinical tests is required to validate our results. 

## 5. Conclusions

To our knowledge, this is the first study to report that Hsp 90, pAkt and GHRH-R in pretreatment tumor samples are possible molecular predictive markers of decreased OS in locally advanced rectal cancer patients. Furthermore, GHRH-R and Hsp90 are independent prognostic factors of 10-year overall survival, and pAkt may have an independent predictive role as well.

We also showed that the intensity levels of pAkt and GHRH-R expression were prognosticators of the time to metastasis. This finding as well as our previous finding that the expression of GHRH-R was an independent prognostic factor for the response to therapy [12], implies that GHRH-R could play a particularly important role as a molecular biomarker. 

A relevant point in our study is the particularly long follow-up period of 10 years, which shows the long-term influence of the investigated biomarkers on the patient outcome. In addition, our report may have novel implications for further research by complementing the existing data on potential biomarkers of rectal cancer and providing a basis for further research involving larger patient populations. Finally, our findings may be of potential practical clinical value. The preferred characteristics of biomarkers include their easy and noninvasive acquisition from the patients, as well as their availability and low expense. Detecting the expression of biomarker proteins using immunohistochemistry could be an easy, cost-effective way of determining prognosis and potentially even planning therapy, since the method is available in medical centers that routinely diagnose and treat patients with cancer, and no additional sampling is required of the patient, since a biopsy is automatically taken for histopathological testing.

Thus, GHRH-R and Hsp90 appear to be promising biomarkers in locally advanced rectal cancer. However, further research, including larger validated studies, is needed to confirm our results.

## Figures and Tables

**Table 1:** Patient and tumor characteristics.

Factor	N = 109
Age (years)	
≤60 >60	60 (55%) 49 (45%)
Sex	
Male Female	55 (50%) 54 (50%)
Clinical T stage	
cT2 cT3 cT4	4 (4%) 93 (85%) 12 (11%)
Clinical N stage	
cN0 cN1-2	41 (38%) 68 (62%)
Distance from AV (cm)	
<5 5–10 >10	34 (31%) 43 (40%) 32 (29%)
Time to surgery (weeks)	
≤7 >7	64 (59%) 45 (41%)
AV: anal verge	

**Table 2:** Immunhistochemical expression of proteins in pretreatment tumor specimens.

Markers	Immunhistochemical Expression			
	Low Intensity		High Intensity	
	0	+	++	+++
SOUL	44 (40%)		65 (60%)	
Hsp 16.2	50 (46%)		59 (54%)	
Hsp 90	36 (33%)		73 (67%)	
p-Akt	26 (24%)		83 (76%)	
GHRH-R	36 (33%)		73 (67%)	

**Table 3:** Relationship between protein expression and the histopathological response to NRCT (n = 109).

	Markers	Case No (*n* = 109)	Good Response (*n* = 52)	Poor Response (*n* = 57)	*p*
**SOUL**	Low intensity	44 (40%)	20 (18%)	24 (22%)	0.699
	High intensity	65 (60%)	32 (30%)	33 (30%)	
**Hsp 16.2**	Low intensity	50 (46%)	28 (26%)	22 (20%)	0.111
	High intensity	59 (54%)	24 (22%)	35 (32%)	
**HSP90**	Low intensity	36 (33%)	20 (18%)	16 (15%)	0.249
	High intensity	73 (67%)	32 (30%)	41 (37%)	
**P-AKT**	Low intensity	26 (24%)	12 (11%)	14 (13%)	0.856
	High intensity	83 (76%)	40 (37%)	43 (39%)	
**GHRH-R**	Low intensity	36 (33%)	22 (20%)	14 (13%)	**0.049**
	High intensity	73 (67%)	30 (28%)	43 (39%)	

**Table 4:** Relationship between clinical parameters and the histopathological response to NRCT (n = 109).

	Clinical Factor	Case No (*n* = 109)	Good Response (*n* = 52)	Poor Response (*n* = 57)	*p*
**Age (years)**	≤60	60 (55%)	30 (27.5%)	30 (27.5%)	0.596
	>60	49 (45%)	22 (20%)	27 (25%)	
**Sex**	Male	55 (50%)	28 (26%)	27 (25%)	0.499
	Female	54 (50%)	24 (22%)	30 (27%)	
Clinical T stage *	cT2	4 (4%)	4 (4%)	0 (0%)	0.068
	cT3	93 (85%)	44 (40%)	49 (45%)	
	cT4	12 (11%)	4 (4%)	8 (7%)	
**Clinical N stage**	cN0	41 (%)	18 (17%)	23 (21%)	0.537
	cN1–2	68 (%)	34 (31%)	34 (31%)	
**Distance from AV (cm)**	<5	34 (31%)	14 (13%)	20 (18%)	0.384
	5–10	43 (39%)	24 (22%)	19 (17%)	
	>10	32 (30%)	14 (13%)	18 (17%)	
**Time to surgery (weeks)**	≤7	64 (59%)	23 (21%)	41 (38%)	**0.003**
	>7	45 (41%)	29 (27%)	16 (14%)	

Note: * 2 cells (33.3%) have expected counts less than 5. The statistical analysis was performed with a chi-square test, level of significance *p* < 0.05.

**Table 5:** Markers showing significant results after the Cox regression analysis of the investigated biological and clinical parameters.

	*B*	*SE*	*Sig.*	*Exp(B)*	*95.0% CI for Exp(B)*	
					Lower	Upper
**Hsp90**	**−0.928**	**0.332**	**0.005**	**0.395**	**0.206**	**0.757**
**GHRH-R**	−2.015	0.562	**0.000**	**0.133**	**0.044**	**0.401**
p-Akt	−0.666	0.470	0.157	0.514	0.205	1.291
Response	0.321	0.284	0.259	1.378	0.789	2.406

## Data Availability

The datasets used and analyzed during the current study are available from the corresponding author on reasonable request.

## Supplementary Materials

The following supporting information can be downloaded at: https://www.mdpi.com/article/10.3390/biomedicines11030719/s1. Supplementary Table S1. The relationships between pretreatment proteins, clinical parameters and OS using the log-rank test.

## Abbreviations

GHRH-R	Growth-hormone-releasing hormone receptor
Hsp	Heat shock protein
LMR	Lymphocyte-to-monocyte ratio
NRCT	Neoadjuvant radiochemotherapy
OS	Overall survival
SOUL	Heme-binding protein 2
TRG	Tumor regression grade
