# Supplementary material for: Expression Levels of GHRH-Receptor, pAkt and Hsp90 Predict 10-Year Overall Survival in Patients with Locally Advanced Rectal Cancer
Source: Biomedicines. 2023 Feb 27;11(3):719. doi: 10.3390/biomedicines11030719 (PMC10045547; doi:10.3390/biomedicines11030719)
Supplement: Supplementary file 1 [file biomedicines-11-00719-s001.zip › biomedicines-2223362-supplementary.pdf]

Supplementary Table S1: The relationship between pre-treatment proteins, clinical parameters and OS using the log-rank test

		Mean[a]	
		**Estimate**	**Std. Error**
**GHRH-R**	Low intensity	112.556	4.154
	High intensity	75.781	4.325
	Log-rank (Mantel-Cox) **Sig.: 0.000**		
**Hsp90**	Low intensity	104.722	4.372
	High intensity	79.644	4.639
	Log-rank (Mantel-Cox) **Sig.: 0.004**		
**p-Akt**	Low intensity	113.077	2.828
	High intensity	80.048	4.304
	Log-rank (Mantel-Cox) **Sig.: 0.001**		
**SOUL**	Low intensity	86.237	6.098
	High intensity	88.563	4.460
	Log-rank (Mantel-Cox) Sig.: 0,661		
**HSP 16.2**	Low intensity	89.920	4.831
	High intensity	85.655	5.308
	Log-rank (Mantel-Cox) Sig.: 0,975		
**Responses**	TRG(3,4,5)	80.351	5.199
	TRG(1,2)	95.280	4,849
	Log-rank (Mantel-Cox) **Sig.: 0.029**		
	upper	100.063	4.599

	middle	75.256	5.957
**Tumor localization**	lower	92.529	6.899
	Log Rank (Mantel-Cox) **Sig.: 0.015**		
**Gender**	male	82.255	4.921
	female	93.704	5.165
	Log Rank (Mantel-Cox) Sig.: 0.057		
**Time to surgery**	<= 7 week	88.508	4.888
	> 7 week	85.636	5.450
	Log Rank (Mantel-Cox) Sig.: 0.568		

[a] Estimation is limited to the largest survival time if it is censored

Supplementary material to the following manuscript:

Expression levels of GHRH-Receptor, pAkt and Hsp90 predict 10-year overall survival in patients with locally advanced rectal cancer
